# Containing anti-PLA2R IgG antibody induces podocyte injury in idiopathic membranous nephropathy

**DOI:** 10.1080/0886022X.2023.2271986

**Published:** 2023-10-31

**Authors:** Ying Zhang, Ping Chen, Baobao Wang, Xueqing Tang, Yong Wei, Wei Cao, Lijun Tang, Zunsong Wang, Na Zhao

**Affiliations:** a Department of Nephrology, The First Affiliated Hospital of Shandong First Medical University & Shandong Provincial Qianfoshan Hospital, Jinan, China; b Shandong Provincial Key Laboratory for Rheumatic Disease and Translational Medicine, Jinan, China; c Nephrology Research Institute of Shandong Province, Jinan, China

**Keywords:** Anti-PLA2R antibody, IgG antibody, idiopathic membranous nephropathy, podocyte injury, in vitro

## Abstract

**Background:**

Idiopathic membranous nephropathy is widely recognized as an autoimmune kidney disease that is accompanied by the discovery of several autoantibodies, and the antibody subclass in the circulation of patients with iMN is mainly IgG. However, the direct pathogenic effect of the containing anti-PLA2R IgG antibody on podocytes is not clear.

**Method:**

A protein G affinity chromatography column was used to purify serum IgG antibodies. Containing anti-PLA2R IgG antibodies from iMN patients and IgG from healthy controls were also obtained. Based on the established *in vitro* podocyte culture system, purified IgG antibodies from the two groups were used to stimulate podocytes, and the expression of essential podocyte proteins (podocin), the levels of inflammatory cytokines in the cell supernatant, cytoskeletal disorders, and podocyte apoptosis were analyzed.

**Results:**

Compared with that in the normal IgG group, the expression of podocin and podocin mRNA was reduced (*p* = 0.016 and *p* = 0.005, respectively), the fluorescence intensity of podocin on the surface of podocytes was reduced, the cytoskeleton of podocytes was disordered and reorganized, and the ratio of podocyte apoptosis was increased in the iMN group (*p* = 0.008).

**Conclusion:**

The containing anti-PLA2R IgG antibody might have a direct damaging effect on podocytes in idiopathic membranous nephropathy.

## Introduction

Membranous nephropathy (MN) is the most common pathological form of adult nephrotic syndrome. Approximately 75-80% of patients do not have a secondary cause, which is defined as primary membranous nephropathy (pMN) and is also known as idiopathic membranous nephropathy (iMN) [1–3]. iMN is widely recognized as an autoimmune kidney disease. In the past 10 years, several target autoantigens have been discovered and confirmed to be specific target antigens of human iMN [4–6], including neutral endopeptidase(NEP), phospholipase A2 receptor, thrombospondin type-1 domain-containing 7 A (THSD7A), exostatin/exostatin 2 (EXT1/EXT2), neuronal epidermal growth factor-like 1 protein (NELL-1), semaphorin-3b (Sema-3B), neural cell adhesion molecule 1 (NCAM-1), high-temperature recombinant protein A1 (HTRA1), and protocadherin 7(PCDH7) [6–12]. Recent studies have confirmed that nearly 70% of iMN patients have anti-PLA2R antibodies in their serum [4], and the proportion of other autoantibodies is low [5, 6]. Because of the heterogeneity of the clinical manifestations, treatment response, and prognosis of iMN, it is especially important to explore its pathogenic mechanism.

The discovery of anti-PLA2R antibodies has great clinical significance for the diagnosis and treatment of iMN. Studies have shown that the level of anti-PLA2R antibodies in peripheral blood is closely related to proteinuria remission in iMN patients [13–15]^,^ and these findings suggest that anti-PLA2R antibodies may be involved in the occurrence and development of iMN. The antibody subclass in the circulation of patients with iMN is mainly IgG. However, the direct pathogenic effect of the containing anti-PLA2R IgG antibody on podocytes is not clear.

Podocytes are the innate cells in the glomerulus. They are mainly involved in forming the glomerular filtration barrier, maintaining the normal opening of capillary loops, synthesizing the basement membrane matrix, secreting cytokines, and regulating endothelial cell functions [16, 17]. Podocyte damage is a key factor in the progression of many diseases [18]. Podocyte injury generally involves foot process fusion, a decrease in the number of podocytes, and apoptosis, which leads to the destruction of the glomerular filtration barrier and high levels of proteinuria [19]. Foot process skeleton protein reorganization is an important cause of foot process fusion [20]. Whether containing anti-PLA2R antibodies can cause podocyte damage in patients with iMN needs to be investigated.

To further clarify the role of IgG autoantibody in the pathogenesis of iMN, containing anti-PLA2R IgG antibodies were purified to determine the direct damaging effect on podocytes *in vitro*.

## Materials and methods

### Materials

VEGF-Aand TGFβ1 ELISA kits were obtained from Labscience (Wuhan, China). The total RNA extraction kit was purchased from Tiangen Biotech Co. Ltd. (Beijing, China), and the reverse transcriptase cDNA synthesis kit and SYBR Green PCR Master Mix assay were purchased from Takara Bio (Beijing, China).GAPDH rabbit polyclonal antibodies were purchased from EnoGene (E12-052). Antibodies against podocin were obtained from Abcam (ab50339). FITC phalloidin was purchased from Solarbio (Beijing, China). The β-actin primers were F CATGTACGTTGCTATCCAGGC and R CTCCTTAATGTCACGCACGAT (Bosune, China). The podocin primers were TTTTCCATCTGGTTCTGCGT and RCATGTCTTTGGTCACGATCTCA (Biosune, China). The Annexin V-FITC/PI Apoptosis Detection Kit was obtained from Elabscience (Wuhan, China).

### Purification of containing anti-PLA2R IgG autoantibody *in vitro*

Blood samples were collected from seven patients who were pathologically diagnosed with iMN, did not use immunosuppressants or hormones, had anti-PLA2R antibodies> 20 RU/ml and were anti-THSD7A antibody-negative on the day of admission. Blood samples from 4 healthy volunteers served as normal controls. The blood samples were stored at −80 °C. Containing anti-PLA2R IgG antibodies were purified from the sera of the seven iMN patients and four healthy controls using a HiTrap Protein G column (Swedish) on an AKTA-FPLC system(GE Biosciences, South San Francisco, CA, USA).

### Cell culture

Human podocytes were provided by Prof. FanYi (Qilu Hospital, Cheeloo College of Medicine, Shandong University) and were cultured at 37 °C, centrifuged at 1000 rpm for 3 min, and discarded, and 10% fetal bovine serum in RPMI 1640 culture solution was added. After being cultured in a 33 °C incubator with 5% CO_2_, the cells were transferred to a 37 °C incubator for differentiation for 2 weeks. Before the experiment, the cells were incubated with serum-free RPMI 1640 medium for 12 h.

The experimental groups included the iMN containing anti-PLA2R IgG antibody group (iMN IgG), healthy human IgG (normal IgG) group, and blank control group (control). According to the time-response curve *in vitro* (Supplemental Figure), containing anti-PLA2R IgG antibodies(200 µg/ml) and healthy human IgG (200 µg/ml) was added and incubated for 72h at 37 °C, 8% healthy serum was added to each group, three wells were set up, and the experiment was repeated three times.

### Analysis of VEGF-a and TGF-β1 levels

After 72 h of incubation, podocyte supernatant was collected, and VEGF-A and TGF-β1 levels were analyzed using commercial ELISA kits (Elabscience, China).

### Western blot analysis of purified containing anti-PLA2R IgG antibodies and podocyte-specific podocin

Western blotting was used to show that the purified serum IgG contained anti-PLA2R antibodies. Briefly, purified IgG from iMN patients was separated by 10% SDS–PAGE for 2 h and transferred to a polyvinylidene fluoride membrane for 2h. Target bands were incubated with rabbit anti-PLA2R1 antibodies (Sigma). Total podocyte proteins were extracted after the cells were incubated for 72 h with purified IgG. Podocyte lysate was separated by 5–10% SDS–PAGE for 2 h and transferred to a polyvinylidene fluoride membrane for 2h. Target bands were incubated with rabbit anti-podocin and rabbit anti-GAPDH antibodies overnight at 4 °C. After being washed, the target bands were incubated with horseradish peroxidase–conjugated IgG, and the blots were visualized with autoradiographic film using electro chemiluminescence plus western blotting detection system (GE Healthcare, Piscataway, USA).

### Real-time quantitative PCR analysis of podocin

Total RNA was extracted from podocyte samples using an RNA isolation kit according to the manufacturer’s instructions and reverse transcribed using a reverse transcriptase cDNA synthesis kit. Podocyte expression of podocin was examined using the SYBR Green PCR Master Mix assay with the following thermal cycling conditions: 95 °C for 5 s, followed by 40 cycles of 5 s at 95 °C and 34 s at 60 °C. Relative gene expression levels were calculated as 2^-ΔΔCt^ using GAPDH as the reference gene.

### Immunofluorescence staining of podocin and the cytoskeleton

The cells were fixed with 4% paraformaldehyde for 10 min and permeabilized with 0.1% Triton X-100 for 5 min. The cells were then stained with anti-podocin antibodies overnight or FITC-labeled phalloidin for 1 h. After being washed, the phalloidin-stained cells were imaged using an inverted fluorescence microscope (LEICA, Germany). TRITC-labeled goat anti-rabbit IgG was diluted in PBS and added to the anti-podocin antibody-stained cells. The stained samples were examined using an inverted fluorescence microscope.

### Podocyte apoptosis detection

The percentage of apoptotic cells in the different groups was detected using an Annexin V-FITC/PI Apoptosis Detection Kit according to the manufacturer’s instructions. Briefly, different groups of podocytes were collected and suspended in a binding buffer after being centrifuged. The cells were then stained with annexin V-FITC for 5 min and with propidium iodide (PI) for 5 min in the dark. A control tube without annexin V-APC/PI was also prepared. The percentage of apoptotic podocytes was determined by flow cytometry (BD Biosciences).

### Statistical analysis

For data with a nonnormal distribution, the results are expressed as the median (interquartile range, IQR). For normally distributed data, the results are expressed as the mean ± standard deviation (SD). Differences were assessed using a one-way analysis of variance (ANOVA) followed by Tukey’s *post hoc* analysis or Mann–Whitney U tests as indicated. Statistical significance was set at *p* < 0.05. Analyses were performed using the SPSS statistical software package (ver. 24.0).

## Results

### The effect of containing anti-PLA2R IgG on the expression of VEGF-a and TGF-β1

**Figure 1.**
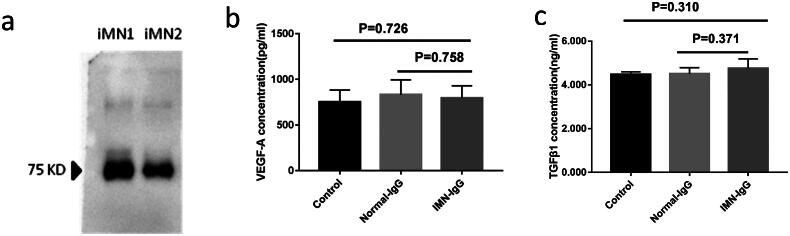
**Expression of VEGF-a and TGFβ in the supernatant of podocytes.** The left panel in Figure 1a shows purified IgG containing the anti-PLA2R antibody, Figure 1b shows the expression level of VEGF-A in podocytes, and the right panel in Figure 1c shows the expression level of TGFβ1 in podocytes. There was no significant difference in the expression of VEGF-A (P = 0.758) and TGFβ1 (P = 0.371) between the containing anti-PLA2R IgG group and the normal IgG group.

Before performing the podocyte assay, purified IgG antibodies were shown to contain anti-PLA2R antibodies by western blotting (Figure 1a). VEGF-A and TGF-β1 are common inflammatory cytokines released by damaged cells, and the levels of these factors in the supernatant were determined by ELISA. After 72 h of incubation, the supernatant was collected for analysis. There were no significant differences in VEGF-A levels between containing anti-PLA2R IgG from iMN patients and normal IgG from healthy controls(832.86 ± 162.54 versus 794.78 ± 135.7 pg/ml, *p* = 0.758) (Figure 1b). Similarly, there was no significant difference in the levels of TGF-β1 between the two groups (4.51 ± 0.28 versus 4.76 ± 0.43 ng/ml, *p* = 0.371) (Figure 1c).

### Effects of containing anti-PLA2R IgG on slit-diaphragm proteins

#### Effect on the expression of podocin

**Figure 2.**
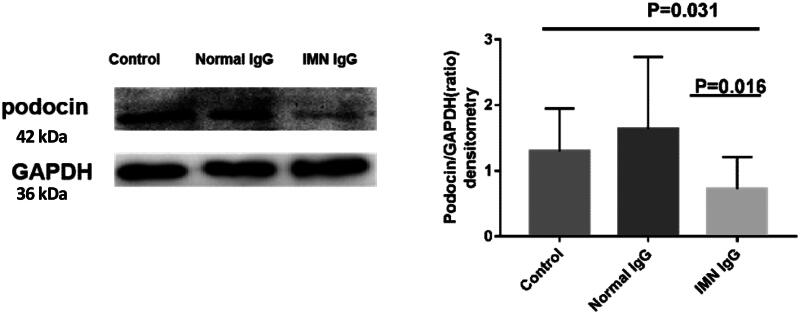
**Podocin protein expression in podocytes.** Containing anti-PLA2R IgG group was stimulated for 72 h, and the expression of podocin was decreased compared with that in the normal IgG stimulation group (P = 0.016).

Since podocin lesions are involved in the pathogenesis of several podocytopathies, we used western blotting to measure the expression of podocin in podocytes. The results showed that the protein expression of podocin in the containing anti-PLA2RIgG group that was treated for 72h was lower than that in the normal IgG group (MFI: median 0.45, IQR 0.27–0.69 versus median 0.97, IQR 0.52–1.61, *p* = 0.016) (Figure 2).

#### Effect on the expression of podocin mRNA

**Figure 3.**
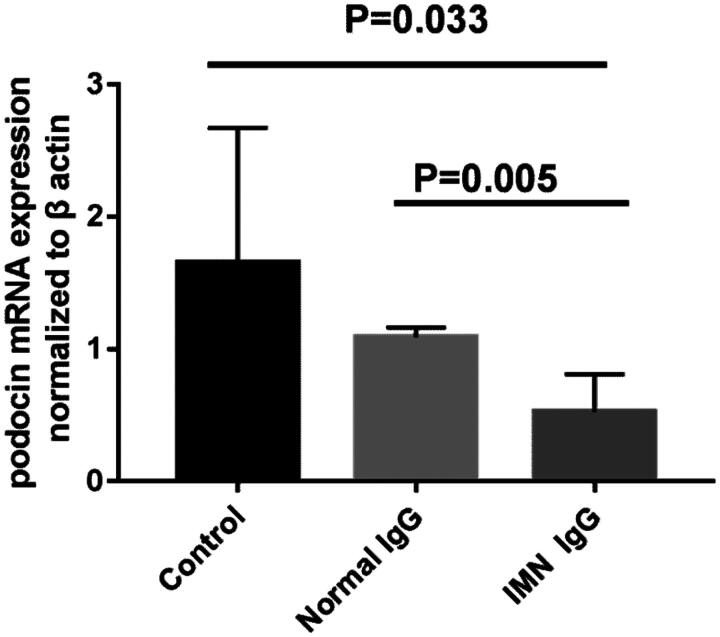
**Podocin mRNA expression in podocytes**. The mRNA expression of podocin in podocytes in the containing anti-PLA2R IgG group was significantly lower than that in the normal IgG stimulation group (P = 0.005).

The mRNA expression of podocin in podocytes was detected by RT–PCR. The results showed that the mRNA expression of podocin in containing anti-PLA2R IgG group that was treated for 72 h was significantly lower than that in the normal IgG group (median 0.49, IQR 0.25-0.79 versus median 1.11 IQR 1.01–1.14,*p* = 0.005) (Figure 3).

#### Effect on the expression of podocin, as determined by immunofluorescence assays

**Figure 4.**
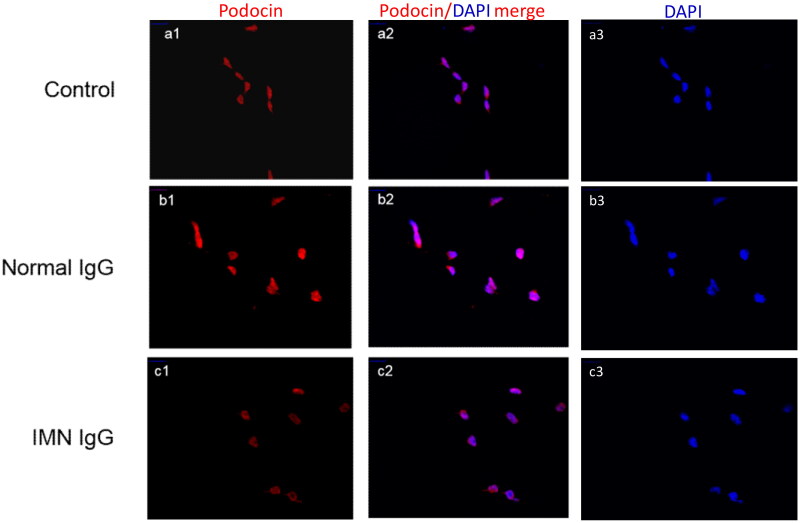
**Fluorescence intensity of podocin in podocytes**. a1, a2 and a3 represent control groups; b1, b2 and b3 represent normal IgG stimulation groups; and c1, c2 and c3 represent containing anti-PLA2R IgG groups. The fluorescence intensity of podocin in the containing anti-PLA2R IgG group was weaker than that in the normal IgG group.

Podocin expression in podocytes was also detected by indirect immunofluorescence analysis. The results showed that the fluorescence intensity of podocin in containing the anti-PLA2R IgG group was weaker than that in the normal IgG group (Figure 4).

### Effect of containing anti-PLA2R IgG on the morphology of the podocyte cytoskeleton

**Figure 5.**
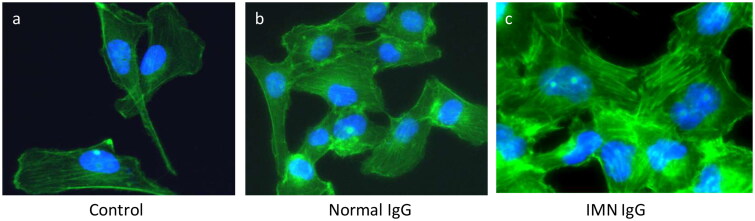
**Immunofluorescence intensity of podocyte cytoskeletal proteins.** a, b and c represent control group, normal IgG stimulation group and containing anti-PLA2R IgG group. The cytoskeleton fibers became shortened, and the arrangement of the fibers was disorganized in the iMN-IgG-treated group. The cytoskeletal structure was disordered after stimulation of containing anti-PLA2R IgG.

Cytoskeleton protein expression was detected by the direct immunofluorescence analysis. F-actin staining by phalloidin showed obvious cytoskeleton disorders; the F-actin fibers were shortened, and the arrangement of the intracellular fibers was disorganized after stimulation with anti-PLA2R IgG (Figure 5).

### Effect of containing anti-PLA2R IgG on podocyte apoptosis

**Figure 6.**
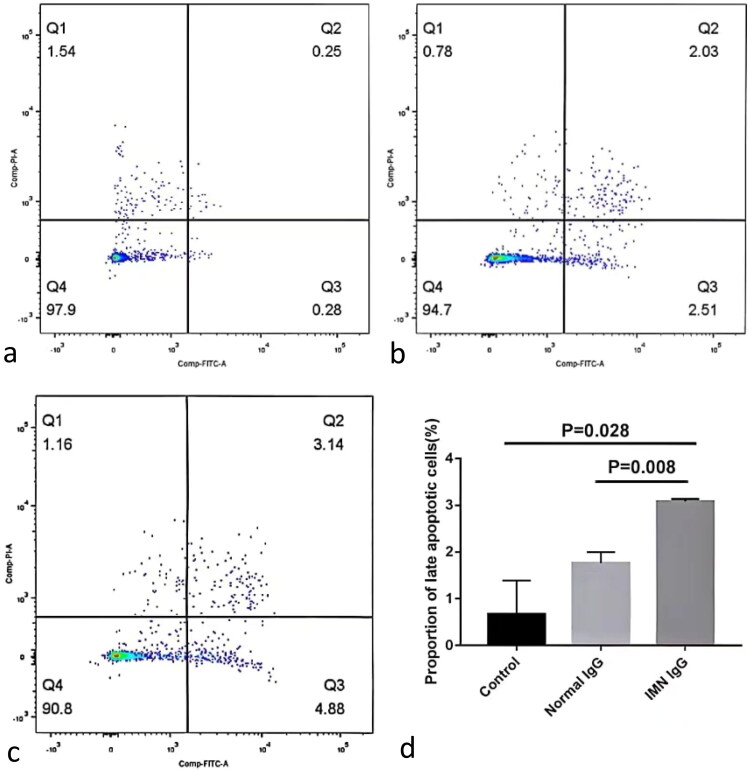
**The level of podocyte apoptosis**. A is the control group, b is the normal IgG group, and c is containing anti-PLA2R IgG group. More apoptosis was observed in containing anti-PLA2R IgG group than in the normal IgG group (P = 0.008).

Flow cytometry was used to detect podocyte apoptosis following IgG stimulation. The results showed that the proportion of podocyte apoptosis in the containing anti-PLA2R IgG group was higher than that in the normal IgG group (*p* = 0.008) (Figure 6).

## Discussion

In this study, we observed that purified containing anti-PLA2R IgG antibodies from iMN patients could reduce the expression of podocin on the surface of podocytes, induce disorder and reorganization of the cytoskeleton, and increase podocyte apoptosis. These findings indicated that containing anti-PLA2R IgG antibodies may cause direct damage to podocytes, which may be independent of the complement system and may not be dependent on the PLA2 receptor on the surface of podocytes. These results provide a new understanding of the pathogenesis of iMN.

Anti-PLA2R antibodies are thought to be a milestone discovery in the pathogenesis of iMN in the past 10 years. Some studies have focused on whether these antibodies have a direct pathogenic role in primary membranous nephropathy. Li Y et al. found that purified anti-PLA2R antibodies could induce podocyte damage independent of the complement system. George et al. found that IgG4 targeted to PLA2R was aberrantly glycosylated and could activate the lectin pathway and induce podocyte injury [21, 22]. Lu [23] found that the integrity of the podocyte cytoskeletal structure was damaged, cell proliferation was decreased, and the apoptosis rate was increased by culturing podocytes with iMN patient serum. Skoberne et al. [24] also showed that patient serum containing anti-PLA2R antibodies interfered with the ability of podocytes to adhere to type IV collagen. However, the damaging effect of purified IgG derived from iMN patients on podocytes was not reported *in vitro*.

The cytoskeleton is a network of protein fibers in eukaryotic cells. Cytoskeleton remodeling is the basis for the increase in albumin permeability [25, 26]. Our experiments confirmed that when human podocytes were exposed to anti-PLA2R IgG antibodies, the cytoskeleton was reorganized. Previous studies have reported that the Rho/ROCK pathway is important for the regulation of cytoskeleton proteins. The integrin/FAK/PI3K pathway is also involved in actin adhesion to the basement membrane [27, 28]. We tried to clarify whether containing anti-PLA2R IgG antibody damaged podocytes directly in this study, and which signal pathways are involved in cytoskeletal rearrangement disorder requires further exploration.

The podocyte split membrane is composed of multiple proteins, including WT1, nephrin, podocin, Neph1, CD2AP, TRPC6, and BKca [29–31]. We selected podocin as the target protein and *NPHS2* as the target gene. Our study showed that after stimulation with IgG extracted from iMN patients, human podocytes and podocin gene and protein expression were significantly decreased. In a study on diabetic nephropathy, serum podocin expression was reduced, and the expression of podocin was significantly inhibited in rat glomeruli stimulated by high glucose [32]. In a study of patients with FSGS, it was found that the expression of podocin in kidney tissue was significantly lower than that in the normal population [33]. Therefore, podocin is involved in the pathogenesis of diabetic nephropathy and FSGS. In a study of iMN, it was found that the decrease in podocin expression was negatively correlated with urine protein levels [34]. In a study of the Heymann nephritis model, the protein expression of podocin decreased, and the decrease in podocin mRNA expression occurred earlier than the decrease in protein expression [35]. In this study, we also observed that podocin protein and mRNA expression decreased after stimulation with IgG derived from iMN patients.

A decrease in the number of podocytes is an important part of the development of MN, and this process can be induced by apoptosis [36, 37]. Studies have shown that the Toll-like receptor 4 (TLR-4)/P2-7 pathway is involved in miR-186-mediated apoptosis [36]. The miR-217/TLR4 pathway can improve podocyte apoptosis by downregulating XIST^37^. The expression of PLA2R in podocytes also participates in the pathogenesis of apoptosis. Yang et al. found that tissue PLA2R staining positively correlated with staining for apoptosis. Dongwei Liu [38] et al. further confirmed that PLA2R overexpression could induce podocyte apoptosis by downregulating the expression of miR-130a-5pin animal experiments. To date, there have only been a few studies on the relationship between containing anti-PLA2R antibodies and cell apoptosis. This study showed that purified containing anti-PLA2R IgG from iMN patients could induce podocyte apoptosis.

In CKD, transforming growth factor-β (TGF-β) is a well-known cytokine that promotes fibrosis and induces podocyte apoptosis [39–41]. Vascular endothelial growth factor A (VEGF-A) is a highly specific pro-vascular endothelial growth factor that is produced and secreted by podocytes and can promote endothelial cell proliferation, migration, and survival [42]. Knockout of podocyte VEGF-A can damage the glomerular filtration barrier, leading to proteinuria and acute renal failure [43]. Overexpression of VEGF-A in adult mice can induce abnormal proteinuria and kidney disease, affecting the structure and function of the glomerular barrier [44]. An increase in VEGF-A overexpression leads to the disappearance of podocytes, the loss of the slit diaphragm, and eventually proteinuria [44, 45]. However, the expression of VEGF-A in iMN patients has rarely been reported. In our study, we found that the expression of TGF-β and VEGF-A did not change significantly after containing anti-PLA2R IgG stimulation of podocytes in iMN patients. These findings suggest that inflammatory cytokines may not act as the core effectors of the development of podocyte lesions.

In summary, we used purified anti-PLA2R IgG antibodies from iMN patients to stimulate podocytes and found that they could cause disorder of the podocyte skeleton, downregulate podocin expression, and directly induce apoptosis in podocytes. Whether the containing anti-PLA2R IgG antibodies include other unknown pathogenic factors or interact with the complement pathway to participate in the pathogenesis of iMN requires further investigation.

## References

[1] Yokoyama H, Taguchi T, Sugiyama H, et al. Membranous nephropathy in Japan: analysis of the Japan renal biopsy registry (J-RBR). Clin Exp Nephrol. 2012;16(4):1–8. doi: 10.1007/s10157-012-0593-7. 22358611

[2] Li LS, Liu ZH. Epidemiologic data of renal diseases from a single unit in China: analysis based on 13,519 renal biopsies. Kidney Int. 2004;66(3):920–923. doi: 10.1111/j.1523-1755.2004.00837.x. 15327382

[3] Alwahaibi NY, Alhabsi TA, Alrawahi SA. Pattern of glomerular diseases in Oman: a study based on light microscopy and immunofluorescence. Saudi J Kidney Dis Transpl. 2013;24(2):387–391. doi: 10.4103/1319-2442.109616. 23538373

[4] Beck LH, Jr., Bonegio RG, Lambeau G, et al. M-type phospholipase A2 receptor as target antigen in idiopathic membranous nephropathy. N Engl J Med. 2009;361(1):11–21. doi: 10.1056/NEJMoa0810457. 19571279 PMC2762083

[5] Tomas NM, Beck LH, Jr., Meyer-Schwesinger C, et al. Thrombospondin type-1 domain-containing 7A in idiopathic membranous nephropathy. N Engl J Med. 2014;371(24):2277–2287. doi: 10.1056/NEJMoa1409354. 25394321 PMC4278759

[6] Sethi S, Debiec H, Madden B, et al. Neural epidermal growth factor-like 1 protein (NELL-1) associated membranous nephropathy. Kidney Int. 2020;97(1):163–174. doi: 10.1016/j.kint.2019.09.014. 31901340

[7] Debiec H, Guigonis V, Mougenot B, et al. Antenatal membranous glomerulonephritis due to anti-neutral endopeptidase antibodies. N Engl J Med. 2002;346(26):2053–2060. doi: 10.1056/NEJMoa012895. 12087141

[8] Sethi S, Madden BJ, Debiec H, et al. Exostosin 1/exostosin 2-associated membranous nephropathy. J Am Soc Nephrol. 2019;30(6):1123–1136. doi: 10.1681/ASN.2018080852. 31061139 PMC6551791

[9] Sethi S, Debiec H, Madden B, et al. Semaphorin 3B-associated membranous nephropathy is a distinct type of disease predominantly present in pediatric patients. Kidney Int. 2020;98(5):1253–1264. doi: 10.1016/j.kint.2020.05.030. 32534052

[10] Caza TN, Hassen SI, Kuperman M, et al. Neural cell adhesion molecule 1 is a novel autoantigen in membranous lupus nephritis. Kidney Int. 2021;100(1):171–181. doi: 10.1016/j.kint.2020.09.016. 33045259 PMC8032825

[11] Al-Rabadi LF, Caza T, Trivin-Avillach C, et al. Serine protease HTRA1 as a novel target antigen in primary membranous nephropathy. J Am Soc Nephrol. 2021;32(7):1666–1681. doi: 10.1681/ASN.2020101395. 33952630 PMC8425645

[12] Sethi S, Madden B, Debiec H, et al. Protocadherin 7-associated membranous nephropathy. J Am Soc Nephrol. 2021;32(5):1249–1261. doi: 10.1681/ASN.2020081165. 33833079 PMC8259689

[13] Oh YJ, Yang SH, Kim DK, et al. Autoantibodies against phospholipase A2 receptor in Korean patients with membranous nephropathy. PLOS One. 2013;8(4):e62151. doi: 10.1371/journal.pone.0062151. 23637987 PMC3637390

[14] Wang J, Xie Q, Sun Z, et al. Response to immunosuppressive therapy in PLA2R- associated and non-PLA2R- associated idiopathic membranous nephropathy: a retrospective, multicenter cohort study. BMC Nephrol. 2017;18(1):227. doi: 10.1186/s12882-017-0636-0. 28693446 PMC5504660

[15] Hoxha E, Harendza S, Pinnschmidt H, et al. M-type phospholipase A2 receptor autoantibodies and renal function in patients with primary membranous nephropathy. Clin J Am Soc Nephrol. 2014;9(11):1883–1890. doi: 10.2215/CJN.03850414. 25267554 PMC4220763

[16] Pavenstadt H, Kriz W, Kretzler M. Cell biology of the glomerular podocyte. Physiol Rev. 2003;83(1):253–307. doi: 10.1152/physrev.00020.2002. 12506131

[17] Scott RP, Quaggin SE. Review series: the cell biology of renal filtration. J Cell Biol. 2015;209(2):199–210. doi: 10.1083/jcb.201410017. 25918223 PMC4411276

[18] Assady S, Benzing T, Kretzler M, et al. Glomerular podocytes in kidney health and disease. Lancet. 2019;393(10174):856–858. doi: 10.1016/S0140-6736(18)33000-9. 30837131

[19] Mathieson PW. The podocyte as a target for therapies–new and old. Nat Rev Nephrol. 2011;8(1):52–56. doi: 10.1038/nrneph.2011.171. 22045242

[20] Ronco P. Proteinuria: is it all in the foot? J Clin Invest. 2007;117(8):2079–2082. doi: 10.1172/JCI32966. 17671644 PMC1934599

[21] Haddad G, Lorenzen JM, Ma H, et al. Altered glycosylation of IgG4 promotes lectin complement pathway activation in anti-PLA2R1-associated membranous nephropathy. J Clin Invest. 2021;131(5):e140453. doi: 10.1172/JCI140453. PMC7919733 33351779

[22] Li Y, Yu J, Wang M, et al. Anti-phospholipase A2 receptor antibodies directly induced podocyte damage in vitro. Ren Fail. 2022;44(1):304–313. doi: 10.1080/0886022X.2022.2039705. 35333675 PMC8959519

[23] Tomas NM, Meyer-Schwesinger C, von Spiegel H, et al. A heterologous model of thrombospondin type 1 domain-containing 7A-associated membranous nephropathy. J Am Soc Nephrol. 2017;28(11):3262–3277. doi: 10.1681/ASN.2017010030. 28814510 PMC5661286

[24] Skoberne A, Behnert A, Teng B, et al. Serum with phospholipase A2 receptor autoantibodies interferes with podocyte adhesion to collagen. Eur J Clin Invest. 2014;44(8):753–765. doi: 10.1111/eci.12292. 24942189

[25] Schell C, Baumhakl L, Salou S, et al. N-wasp is required for stabilization of podocyte foot processes. J Am Soc Nephrol. 2013;24(5):713–721. doi: 10.1681/ASN.2012080844. 23471198 PMC3636796

[26] Wang L, Zhang D, Zheng J, et al. Actin cytoskeleton-dependent pathways for ADMA-induced NF-kappaB activation and TGF-beta high expression in human renal glomerular endothelial cells. Acta Biochim Biophys Sin. 2012;44(11):918–923. doi: 10.1093/abbs/gms077. 23027376

[27] Tian X, Ishibe S. Targeting the podocyte cytoskeleton: from pathogenesis to therapy in proteinuric kidney disease. Nephrol Dial Transplant. 2016;31(10):1577–1583. doi: 10.1093/ndt/gfw021. 26968197 PMC5039341

[28] Lin Y, Rao J, Zha XL, et al. Angiopoietin-like 3 induces podocyte F-actin rearrangement through integrin alpha(V)beta(3)/FAK/PI3K pathway-mediated Rac1 activation. Biomed Res Int. 2013;2013:135608–135608. doi: 10.1155/2013/135608. 24294595 PMC3835706

[29] Holthofer H, Ahola H, Solin ML, et al. Nephrin localizes at the podocyte filtration slit area and is characteristically spliced in the human kidney. Am J Pathol. 1999;155(5):1681–1687. doi: 10.1016/S0002-9440(10)65483-1. 10550324 PMC1866978

[30] Morton MJ, Hutchinson K, Mathieson PW, et al. Human podocytes possess a stretch-sensitive, Ca2+-activated K + channel: potential implications for the control of glomerular filtration. J Am Soc Nephrol. 2004;15(12):2981–2987. doi: 10.1097/01.ASN.0000145046.24268.0D. 15579500

[31] Kim EY, Alvarez-Baron CP, Dryer SE. Canonical transient receptor potential channel (TRPC)3 and TRPC6 associate with large-conductance Ca2+-activated K+ (BKCa) channels: role in BKCa trafficking to the surface of cultured podocytes. Mol Pharmacol. 2009;75(3):466–477. doi: 10.1124/mol.108.051912. 19052171 PMC2645922

[32] Menon R, Mohd Noor FS, Draman CR, et al. A retrospective review of diabetic nephropathy patients during referral to the Sub-urban nephrology clinic. Saudi J Kidney Dis Transpl. 2012;23(5):1109–1114. doi: 10.4103/1319-2442.100972. 22982937

[33] Horinouchi I, Nakazato H, Kawano T, et al. In situ evaluation of podocin in normal and glomerular diseases. Kidney Int. 2003;64(6):2092–2099. doi: 10.1046/j.1523-1755.2003.00303.x. 14633131

[34] Agrawal V, Prasad N, Jain M, et al. Reduced podocin expression in minimal change disease and focal segmental glomerulosclerosis is related to the level of proteinuria. Clin Exp Nephrol. 2013;17(6):811–818. doi: 10.1007/s10157-013-0775-y. 23377573

[35] Stratakis S, Stylianou K, Petrakis I, et al. Rapamycin ameliorates proteinuria and restores nephrin and podocin expression in experimental membranous nephropathy. Clin Dev Immunol. 2013;2013:941893–941898. doi: 10.1155/2013/941893. 24069045 PMC3773418

[36] Sha WG, Shen L, Zhou L, et al. Down-regulation of miR-186 contributes to podocytes apoptosis in membranous nephropathy. Biomed Pharmacother. 2015;75:179–184. doi: 10.1016/j.biopha.2015.07.021. 26382839

[37] Jin LW, Pan M, Ye HY, et al. Down-regulation of the long non-coding RNA XIST ameliorates podocyte apoptosis in membranous nephropathy via the miR-217-TLR4 pathway. Exp Physiol. 2019;104(2):220–230. doi: 10.1113/EP087190. 30414341

[38] Liu D, Liu F, Wang X, et al. MiR-130a-5p prevents angiotensin II-induced podocyte apoptosis by modulating M-type phospholipase A2 receptor. Cell Cycle. 2018;17(21–22):2484–2495. doi: 10.1080/15384101.2018.1542901. 30394845 PMC6342077

[39] Gruden G, Perin PC, Camussi G. Insight on the pathogenesis of diabetic nephropathy from the study of podocyte and mesangial cell biology. Curr Diabetes Rev. 2005;1(1):27–40. doi: 10.2174/1573399052952622. 18220580

[40] Wolf G, Ziyadeh FN. Cellular and molecular mechanisms of proteinuria in diabetic nephropathy. Nephron Physiol. 2007;106(2):p26–31. doi: 10.1159/000101797. 17570945

[41] Schiffer M, Bitzer M, Roberts IS, et al. Apoptosis in podocytes induced by TGF-beta and Smad7. J Clin Invest. 2001;108(6):807–816. doi: 10.1172/JCI200112367. 11560950 PMC200928

[42] Simon M, Grone HJ, Johren O, et al. Expression of vascular endothelial growth factor and its receptors in human renal ontogenesis and in adult kidney. Am J Physiol. 1995;268(2 Pt 2):F240–250. doi: 10.1152/ajprenal.1995.268.2.F240. 7864162

[43] Veron D, Villegas G, Aggarwal PK, et al. Acute podocyte vascular endothelial growth factor (VEGF-A) knockdown disrupts alphaVbeta3 integrin signaling in the glomerulus. PLOS One. 2012;7(7):e40589. doi: 10.1371/journal.pone.0040589. 22808199 PMC3396653

[44] Veron D, Reidy KJ, Bertuccio C, et al. Overexpression of VEGF-A in podocytes of adult mice causes glomerular disease. Kidney Int. 2010;77(11):989–999. doi: 10.1038/ki.2010.64. 20375978

[45] Baelde HJ, Eikmans M, Lappin DW, et al. Reduction of VEGF-A and CTGF expression in diabetic nephropathy is associated with podocyte loss. Kidney Int. 2007;71(7):637–645. doi: 10.1038/sj.ki.5002101. 17264876

